# Effectiveness and safety of a newly designed self-assembling gel in the treatment of endoscopic submucosal dissection-induced gastric ulcer: A multicenter randomized controlled trial

**DOI:** 10.3389/fphar.2022.1002381

**Published:** 2022-12-01

**Authors:** Meng Li, Xiaoliang Jin, Xinxin Zhou, Guochun Lou, Feng Ji, Liangjing Wang, Haifeng Jin, Xuan Huang, Jing Zhao, Haibiao Bao, Liang Huang, Changpei Shi, Bo Jin, Hanti Lu, Bin Lyu

**Affiliations:** ^1^ Department of Gastroenterology, First Affiliated Hospital of Zhejiang Chinese Medical University, Hangzhou, China; ^2^ First Clinical Medical College, Zhejiang Chinese Medical University, Hangzhou, China; ^3^ Department of Gastroenterology, First Affiliated Hospital of Zhejiang University, Hangzhou, China; ^4^ Department of Gastroenterology, Second Affiliated Hospital of Zhejiang University, Hangzhou, China

**Keywords:** artificial ulcer, endoscopic submucosal dissection, gel, self-assembled, ulcer healing

## Abstract

**Objectives:** To evaluate the effectiveness and safety of a newly designed self-assembling gel in treating ESD-induced gastric ulcers in patients.

**Methods:** This open-label, multicenter, randomized controlled trial enrolled patients who underwent ESD between September 2020 and May 2021. Patients were randomized (1:1) to receive the gel (applied to cover the entire ulcer bed under endoscopic guidance immediately after ESD; gel group) or not (control group). The primary outcome was the ulcer healing rate at 28 days. And the secondary outcomes were the delayed bleeding, changes in the ulcer stage, and adverse events.

**Results:** Finally, 125 patients (mean age, 63.7 years; 70 [56.0%] males) were enrolled. The ulcer healing rate was higher in the gel group than in the control group at 28 days (96.9 ± 4.1% vs. 94.7 ± 5.0%; *p =* 0.001). The ulcer reduction rate at 28 days differed significantly (*p <* 0.001) between ulcers with majority gel coverage (99.8%), ulcers with minority gel coverage (96.2%), and ulcers with no gel coverage (98.0%). Delayed bleeding was found in 1/63 gel-treated patients (1.6%) versus 5/62 controls (8.1%). A1-stage ulcers were found in 16/63 patients in the gel group versus 44/62 patients in the control group (25.4% vs. 71.0%, *p <* 0.001) at 3–5 days.

**Conclusion:** The newly developed self-assembling gel was safe and effective in accelerating gastric ulcer healing in patients after ESD.

**Clinical Trial Registration:** UMIN Clinical Trials Registry System (registration number, ChiCTR2100052935).

## Introduction

Gastric cancer is the sixth most common malignancy worldwide and the second leading cause of cancer-related death (Chen, 2015; Bray et al., 2018). Upper gastrointestinal tract cancers are more common in Eastern Asia than in western countries (Chen, 2015; Bray et al., 2018). Advances in endoscopic techniques over recent decades improved the detection of gastric cancer (Waddingham et al., 2021), and the 5-year survival rate for early-stage gastric cancer now exceeds 80% (Tan, 2019). Endoscopic submucosal dissection (ESD) is a minimally invasive technique that allows the en-bloc resection of early-stage gastrointestinal tract malignancies (Draganov et al., 2019). ESD is an effective and reasonably safe method of resecting early-stage gastric cancer (Ahn and Jung, 2013; Kim et al., 2013) and is used widely as a first-line option for the surgical treatment of superficial gastric lesions (Pimentel-Nunes et al., 2015; NCCN Clinical Practice Guidelines in, 2022).

Currently, there are no standardized therapies for ESD-induced large iatrogenic ulcers in the stomach. Delayed bleeding is an important complication of ESD and occurs in 5% of the cases, even under standard proton pump inhibitor (PPI) therapy (Uedo et al., 2007; Goto et al., 2012; Toyokawa et al., 2012). Although PPIs are commonly used to treat ESD-induced ulcers, the healing rates at 4 weeks are only 15%–36% for PPI monotherapy and 19%–68% for PPI in combination with a muco-protective agent (Kato et al., 2010; Kobayashi et al., 2012; Takayama et al., 2013). Agents such as vonoprazan (a potassium-competitive acid blocker) have been tried but were not superior to PPIs (Kim et al., 2019). The combination of a PPI and muco-protective agent is considered insufficient for iatrogenic gastric ulcers that are large or exhibit severe gastric atrophy (Fujiwara et al., 2011; Kobayashi et al., 2012).

Methods conferring mechanical protection, such as polyglycolic acid (PGA) sheets or bio-sheets, have been developed to accelerate artificial ulcer healing or reduce post-ESD adverse events (Kwon et al., 2015; Sakaguchi et al., 2015). A previous study developed a new gel comprised of two different biocompatible and pyrogen-free materials; this new self-assembling gel was demonstrated to adhere successfully to the wound’s surface after ESD, accelerating the healing of post-ESD ulcers and enhance epithelial regeneration in an experimental porcine model (Li et al., 2021). This study aimed to evaluate the effectiveness and safety of this gel in the treatment of ESD-induced gastric ulcers in patients.

## Methods

### Study design

This open-label, blinded endpoint, multicenter, randomized controlled trial was conducted in accordance with the tenets of the Declaration of Helsinki and approved by the institutional review board of First Affiliated Hospital of Zhejiang Chinese Medical University (2020-Q-004-01). Signed informed consent was provided by each patient before enrollment. The trial is registered in the UMIN Clinical Trials Registry System (registration number, ChiCTR2100052935) and carried out under the supervision of the Drug Administration of Zhejiang Province.

### Patients

The study enrolled patients scheduled for ESD of gastric adenoma or early gastric cancer at the First Affiliated Hospital of Zhejiang Chinese Medical University, First Affiliated Hospital of Zhejiang University, and Second Affiliated Hospital of Zhejiang University between September 2020 and May 2021. The inclusion criteria were 1) 18–80 years of age and 2) pathological diagnosis of gastric adenoma or cancer treatable by ESD, with lesion size ≥ 2 cm. The exclusion criteria were 1) a history of active infection or severe systemic disease, 2) severely impaired liver, kidney, or cardiopulmonary function, 3) a history of coagulation disorders, 4) pregnant or breastfeeding women, 5) systemic administration of corticosteroids in the previous 6 weeks, 6) anticoagulants or antiplatelets administered parenterally, 7) known hypersensitivity to any gel constituent, or 8) patients with multiple lesions.

### Randomization and blinding

An independent study manager developed a randomization sequence using the block design method (block size of 4). Blocks were randomized based on a random-number table generated by SAS 9.3 (SAS Institute, United States). This study was open-label for the patients and medical workers who administered the treatment. The endoscopy experts who were not involved in the ESD procedure, the statisticians who carried out the data analysis, and the medical staff who monitored the patients for adverse events were blinded to the grouping. Independent statisticians carried out the data analysis, contributed to the experimental design and implementation, performed sample blinding, managed the data, and completed a summary statistical report.

### Intervention

The eligible patients were randomized into the gel or control group. The patients in both groups were admitted the day before ESD. The ESD procedure was carried out by experienced endoscopists, with the patient sedated by continuous propofol infusion. ESD was performed according to standard methods (Committee et al., 2015), and patients in both groups underwent conventional endoscopic hemostasis for the wound.

**FIGURE 1:**
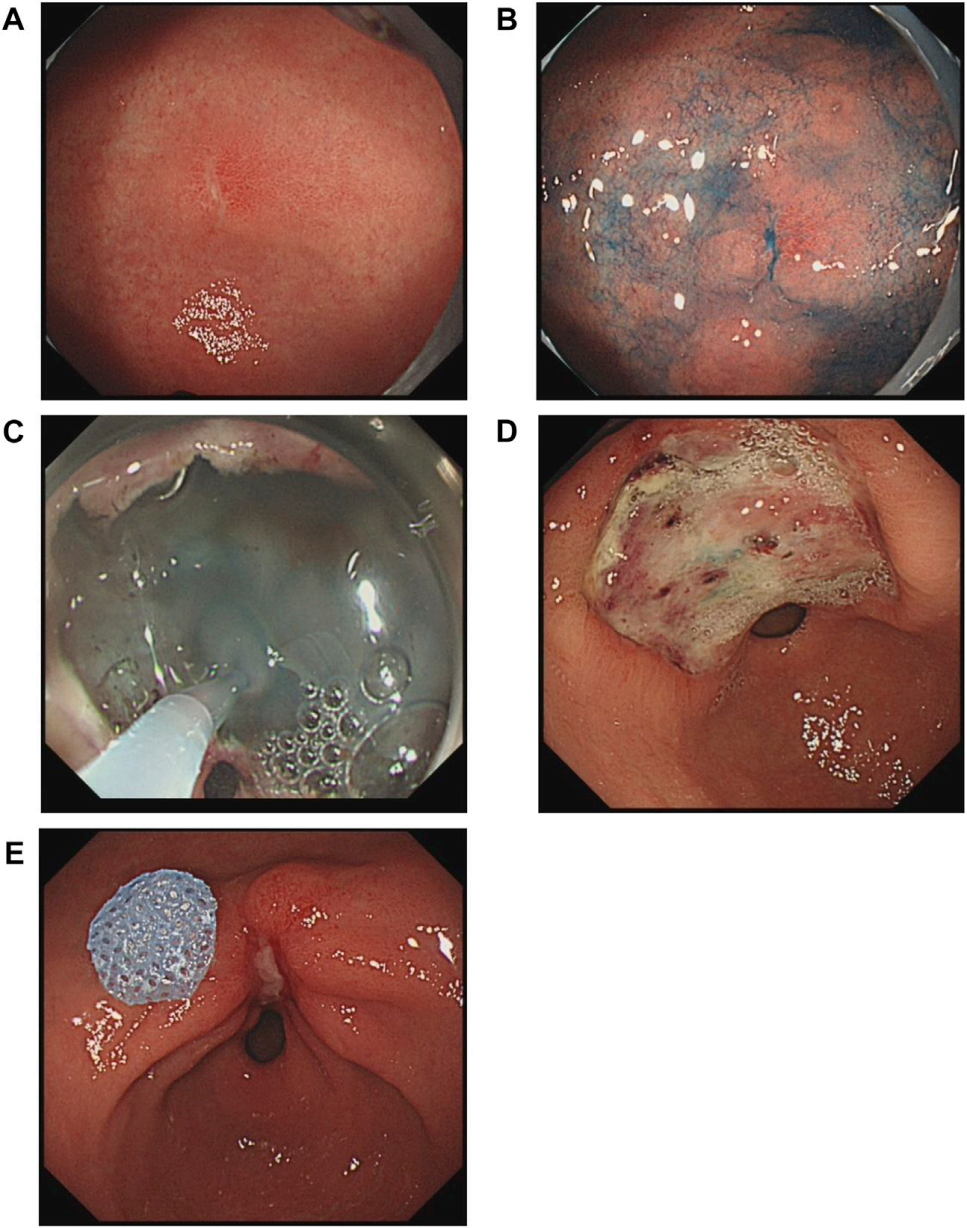
Changes in a gastric antral ulcer induced by endoscopic submucosal dissection (ESD) in a gel-treated patient. **(A)**, An early-stage cancer on the lesser curvature of the antrum. **(B)**, Endoscopic image after spraying with indigo-carmine dye. **(C)**, Application of gel to the ulcer immediately after ESD. **(D)**, The ulcer was in the active stage according to the Sakita and Fukutomi classification at 3 days after ESD. **(E)**, The healing stage was observed at 28 days after ESD.

The patients in the gel group were treated with the novel gel immediately following ESD. The gel is comprised of two proprietary constituents that are nontoxic and biocompatible. The gel used in this study was self-designed. It consists of two components (a colloid solution and a fixative solution). The colloid solution consists of sodium alginate, polylysine, magnesium lithium silicate (Laponite-XLG XR), NaCl, and purified water. Since it is sensitive to acid and calcium, the fixative solution contains CaCl_2_ and purified water. After mixing, the two solutions rapidly self-assemble into a solid film made of a complex network of polysaccharides and amino acids, which further solidifies in the presence of gastric acid. This gel has already been registered with the National Medical Products Administration of China (20222140160) and is now commercially available from Yingjian Biotechnology Co., Ltd. The cross-linked gel network attaches to the floor of an ulcer *via* ionic bonds. Indigo-carmine dye was mixed with the gel during its application to visualize whether the ulcer surface was fully covered and to allow clear observation of the gel coverage during follow-up. In this study, 20–30 ml of the colloid solution and 10–20 ml of the fixative solution were used to cover a 2-cm ulcer. After cleaning the ulcer and neighboring region, the ulcer was sprayed first with the colloid solution and then with the fixative solution under endoscopic guidance. Gel solidification occurred within 3–5 min. Images of an ulcer treated with the gel are shown in Figure 1. The ulcers of patients in the control group were not sprayed with any substances.

In patients without signs of hemorrhage, a liquid diet was given on the second postoperative day, and a semi-liquid, fiber-free diet was given from the third postoperative day. All patients received intravenous pantoprazole 40 mg or an equivalent PPI (e.g., omeprazole 40 mg or ilaprazole 10 mg) every 12 h on the first 3–5 days after ESD, followed by oral pantoprazole 40 mg/day or an equivalent PPI (e.g., rabeprazole 10 mg, or omeprazole 20 mg) on postoperative days 6–28.

## Outcomes

The primary outcome was the ulcer reduction rate at 28 days following ESD, which was calculated as (initial ulcer area-ulcer area)/initial ulcer area × 100%. The initial ulcer area was determined by measuring the resected specimen after fixation using ImageJ 1.47 (National Institutes of Health, United States). The measurement of the ulcer area during follow-up endoscopy was facilitated by placing a 20-mm diameter blue paper disk at the boundary of the ulcer. The initial ulcer area and follow-up ulcer area were determined by two independent medical workers who were blinded to grouping.

The secondary outcomes included delayed bleeding following ESD, ulcer stage, and gel coverage at 3–5 days and 28 days following ESD. Delayed bleeding following ESD was defined as major and minor bleeding. Major bleeding was defined as hematemesis or melena that required endoscopic hemostasis or surgery, while minor bleeding was considered if a patient developed hematemesis, melena, or hemoglobin levels decreasing by ≥ 2 g/dl accompanying blood clots and exposed vessels detected by endoscopy post-ESD excluding the major bleeding. The ulcer stage was assessed endoscopically and classified into one of the following six categories using the method proposed by Sakita and Fukutomi (Sakita, 1972): active (A1 or A2), healing (H1 or H2), or scarring (S1 or S2). The coverage rate of gel adhering to the ulcer surface was defined as majority gel coverage (25%–50% gel coverage), minority gel coverage (1%–25% gel coverage), and no gel coverage.

The adverse events during the hospital stay, including abdominal pain, melena (black stools), fecal occult blood positivity, endoscopic hemorrhage, throat discomfort, rash, fall in hemoglobin level, dizziness, and fever, were assessed each day through interviews and physical examinations.

### Sample size calculation

The pilot study showed that the ulcer reduction rate was 10% higher in the gel group than in the control group, and the standard deviation (SD) was 16% in both groups. Based on the pilot study, it was calculated that 54 patients would be needed for each group (α = 0.025, power of test = 90%). Assuming a dropout rate of 10%, at least 60 patients were included in each group.

### Statistical analysis

Data analysis was performed using SPSS 22.0 (IBM, Armonk, New York, United States). Normally distributed continuous variables were presented as mean ± SD and analyzed using Student’s t-test. Non-normally distributed continuous variables were described as median (range) and analyzed using the Mann-Whitney U-test. Categorical variables were expressed as n (%) and analyzed using Pearson’s chi-squared test. The bleeding curves were analyzed using the Kaplan-Meier method and the Breslow test. Subgroup analysis was used to compare ulcer reduction rates between patients with different gel cover rates. A two-sided *p* < 0.05 was considered statistically significant.

The efficacy analysis was carried out based on the modified intent-to-treat set, excluding individuals who withdrew consent (full analysis set), and further excluded limited participants from the standard ITT set. The efficacy was also assessed in cases fulfilling the treatment protocol (per-protocol set). The safety analysis was performed in individuals administered at least one dose of the study treatment and ESD (safety analysis set). Multiple imputations (MI) were used for missing data.

## Results

### Baseline characteristics of the study participants

**FIGURE 2:**
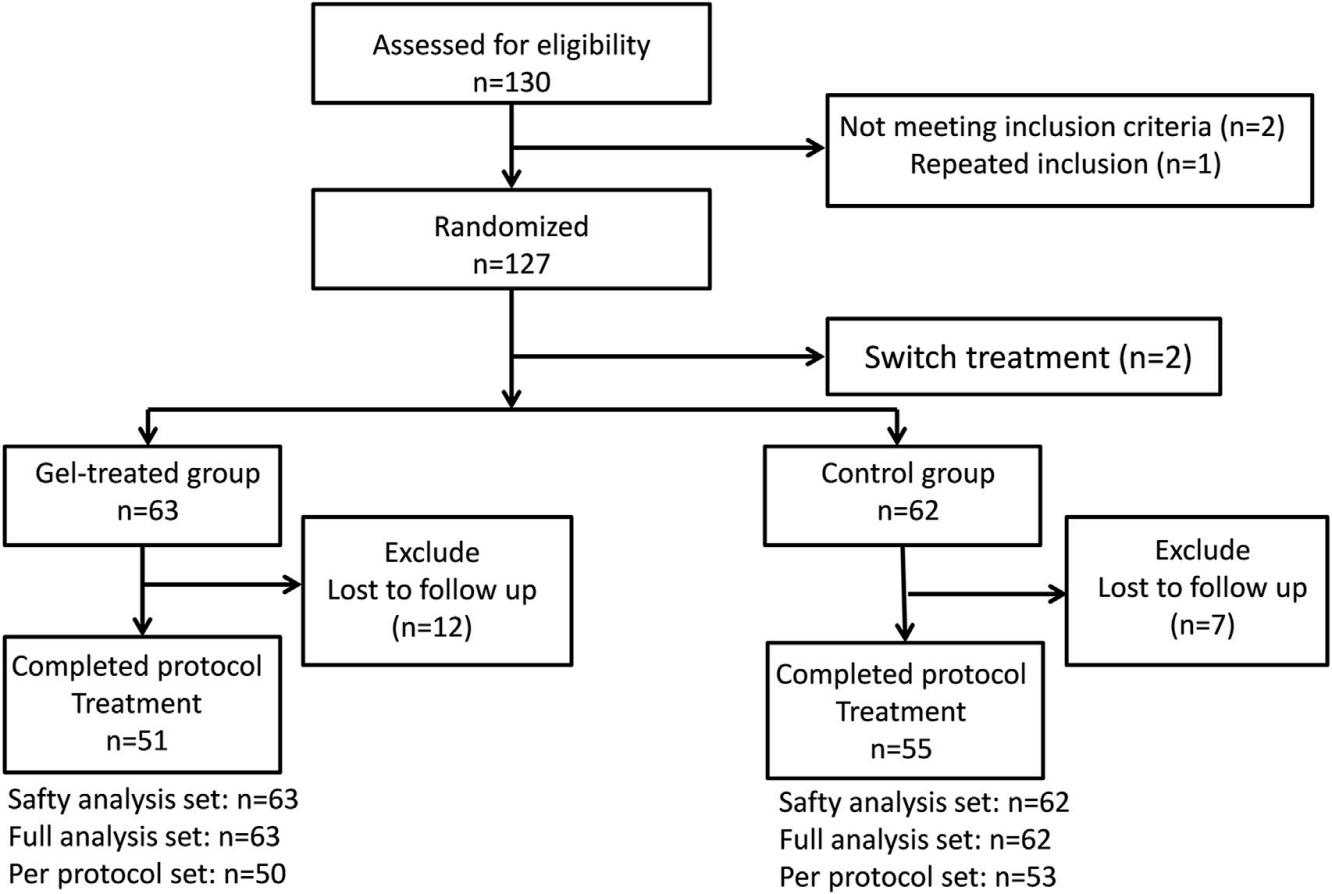
Study flowchart.

Among 130 eligible patients initially screened for this study, two patients were excluded due to lesions <2 cm in size, and one patient was excluded due to repeat inclusion. Further two patients (one from each group) were excluded after randomization because additional hemostasis clipping was required immediately after ESD to treat arteriolar bleeding on the artificial ulcer floor. Considering that hemostasis clipping would lead to a hard-to-measure ulcer area, the two participants did not receive the planned treatment or ulcer measurement according to the study design. Therefore, 125 patients who underwent ESD were included in the study (gel group, *n* = 63; control group, *n* = 62). Seven participants in the control group and 12 in the gel group were lost to follow-up. Thus, 106 participants completed the 28-day study protocol (gel group, *n* = 51; control group, *n* = 55) (Figure 2). The demographic and clinical data at baseline did not differ significantly between the two groups (Table 1, Supplementary Table S1).

**TABLE 1:** Baseline characteristics of the study participants.

	Gel group (*n* = 63)	Control group (*n* = 62)	*p*
Mean age (years), mean ± SD	60.8 ± 12.6	65.2 ± 9.6	0.155
Male, *n* (%)	35 (57.1)	35 (56.5)	>0.999
Body mass index (kg/m^2^), mean ± SD	23.4 ± 2.7	23.5 ± 3.2	>0.999
Current alcohol consumption, *n* (%)	11 (17.5)	10 (16.1)	>0.999
Current smoker, *n* (%)	16 (25.4)	10 (16.1)	>0.999
Diabetes mellitus, *n* (%)	6 (9.5)	7 (11.3)	>0.999
Hypertension, *n* (%)	22 (34.9)	22 (35.5)	>0.999
Tumor location, *n* (%)			0.624
Antrum	36 (57.1)	33 (53.2)	
Angle	11 (17.5)	8 (12.9)	
Upper body	8 (12.7)	8 (12.9)	
Lower body	8 (12.7)	13 (21.0)	
Tumor with scar, *n* (%)	1 (1.3)	1 (1.3)	>0.999
Sample size after fixation (mm^2^), median (IQR)	855.5 (721.5)	750.2 (583.9)	0.371
Procedure time for ESD (min), mean ± SD	55.0 ± 57.0	48.5 ± 49.0	0.859
Injection time for ESD (min), mean ± SD	2.5 ± 1.0	2.5 ± 1.63	0.944
En-bloc resection, *n* (%)	61 (96.8)	59 (95.2)	0.492
Complete resection, *n* (%)	61 (96.8)	59 (95.2)	0.492
PPI used during the first 3–5 days, *n* (%)			0.432
Ilaprazole	7 (4.8)	3 (4.8)	
Omeprazole	28 (44.4)	30 (48.4)	
Pantoprazole	28 (44.4)	29 (46.8)	
PPI used during postoperative days 6–28, *n* (%)			0.649
Omeprazole	21 (33.3)	20 (32.3)	
Rabeprazole	28 (44.4)	26 (41.9)	
Pantoprazole	4 (6.3)	8 (12.9)	
Na	10 (15.9)	8 (12.9)	

IQR, interquartile range; SD, standard deviation. PPI, proton pump inhibitor.

### Ulcer healing at 3–5 days and 28 days after ESD

This primary outcome could not be measured in one participant from the gel group and two participants from the control group due to the folding of the paper disk used as a reference. The ulcer reduction rate at the 28-day follow-up was significantly higher in the gel group than in the control group in the full analysis set (96.9% ± 4.1% vs. 94.7% ± 5.0%, *p =* 0.001) as well as in the per-protocol set (96.8% ± 4.0% vs. 94.5% ± 5.0%; *p =* 0.001; Table 2). The ulcer reduction rate at 28 days was also significantly higher in the gel group than in the control group before MI (Supplementary Table S2).

**TABLE 2:** Ulcer healing at 3–5 days and 28 days after endoscopic submucosal dissection (ESD).

Characteristics	Full analysis set			Per protocol set		
	Control group (*n* = 62)	Gel group (*n* = 63)	*p*	Control group (*n* = 53)	Gel group (*n* = 50)	*p*
Ulcer area (mm^2^), median (IQR)						
Initial	763.4 (574.4)	863.6 (712.6)	0.067	750.2 (494.8)	868.7 (726.9)	0.017
28 days after ESD	34.7 (50.9)	15.4 (52.4)	<0.001	34.7 (50.0)	15.3 (39.3)	<0.001
Ulcer reduction rate at 28 days						
All patients, mean ± SD	94.7± 5.0	96.9 ± 4.1	<0.001	94.5 ± 5.0	96.8 ± 4.0	0.009
No gel coverage at 3–5 days, median (IQR)		98.0 (3.04)			98.0 (3.02)	
Minority gel coverage at 3–5 days, median (IQR)		96.2 (5.14)			96.3 (3.86)	
Majority gel coverage at 3–5 days, median (IQR)		99.8 (0.52)	<0.001		99.8 (1.25)	<0.001
Ulcer stage at 3–5 days, *n* (%)			<0.001			<0.001
A1 stage	44 (71.0)	16 (25.4)		36 (67.9)	13 (26.0)	
A2 stage	17 (27.4)	45 (71.4)		17 (32.1)	35 (70.0)	
H1 stage	1 (1.6)	2 (3.2)		0	2 (4.0)	
Ulcer stage at 28 days, *n* (%)			0.143			0.205
A2 stage	6 (9.7)	6 (9.5)		6 (11.3)	5 (10.0)	
H1 stage	41 (66.1)	38 (60.3)		36 (67.9)	32 (64.0)	
H2 stage	10 (16.1)	5 (7.9)		8 (15.1)	4 (8.0)	
S1 stage	5 (8.1)	14 (22.2)		3 (5.7)	9 (18.0)	
Delayed bleeding, *n* (%)	8 (12.9)	6 (9.5)	0.549	7 (13.2)	5 (10.0)	0.761

Endoscopy performed 3–5 days after ESD demonstrated an A1 stage ulcer in 16 of 63 participants in the gel group versus 44 of 62 patients in the control group (25.4% vs. 71.0%, *p* < 0.001). Ulcer stage classification at the 28-day follow-up exhibited no significant between-group differences in the full analysis and per-protocol sets (Table 2).

Four of the 50 participants in the gel group who underwent endoscopy at the 28-day follow-up declined endoscopy at 3–5 days after ESD. Among the remaining 46 participants in the gel group, six had majority gel coverage, 17 had minority gel coverage, and 23 had no gel coverage, according to the endoscopic images obtained at 3–5 days post-ESD (Table 2). The ulcer reduction rate differed significantly between cases with majority gel coverage, cases with minority gel coverage, and cases with no gel coverage (99.8% vs. 96.2% vs. 98.0%, *p <* 0.001).

### Delayed bleeding after ESD

Major bleeding occurred in only one participant from the control group. The participant presented with hematemesis only 5 h after ESD and needed endoscopic hemoclipping. Minor bleeding occurred in one of the 63 participants in the gel group (1.6%; 95% confidence interval [95%CI], -1.6%–4.8%) and five of the 62 participants in the control group (8.1%; 95%CI, 1.1%–15.0%; Table 2). Four of six participants (one in the gel group and three in the control group) reported hematemesis and melena after ESD. No participants needed a blood transfusion. The total delayed bleeding rates were not significantly different between groups based on the full analysis and per-protocol sets (gel group: 2.0%; 95%CI, -2.2%–6.0%; control group: 7.5%; 95%CI, 0.2%–14.9%). The delayed bleeding rates were also not significantly different between groups before multiple interpolations (Supplementary Table S2).

## Adverse events

During the study period, 62.9% of the participants in the control group reported an adverse event, compared with 68.3% of the gel-treated participants (Table 3). There were no statistically significant differences between the two groups. Most adverse events were mild or moderate in severity (CTCAE grade 1 or 2) (Table 3). There were no significant differences between groups in the results of routine examinations during follow-up, except for diastolic blood pressure (Supplementary Table S1).

**TABLE 3:** Adverse events.

Adverse events, *n* (%)	Control group (*n* = 62)	Gel group (*n* = 63)	*p*
Abdominal pain	10 (16.1)	16 (25.4)	0.271
Black stool	2 (3.2)	4 (6.3)	0.348
Fecal occult blood positive	2 (3.2)	1 (1.6)	0.494
Constipation	1 (1.6)	3 (4.8)	0.316
Oozing bleeding under endoscopy	6 (9.7)	6 (9.5)	>0.99
Throat discomfort	3 (4.8)	1 (1.6)	0.303
Rash	2 (3.2)	1 (1.6)	0.494
Hemoglobin drop >2 g/d	3 (4.8)	0 (0)	0.119
Dizziness	1 (1.6)	0 (0)	0.496
Fever	9 (14.5)	11 (17.5)	0.808

## Discussion

Previous studies reported that administering a PPI in combination with a muco-protective agent achieved ulcer healing rates of only 19%–68% after 28 days (Kato et al., 2010; Kobayashi et al., 2012; Takayama et al., 2013). In the present study, the proportion of S-stage cases was only 8.1% in the control group (full analysis set; Table 2), which is lower than the values of 17.4%–36.4% described previously for PPI-treated patients (Takayama et al., 2013; Kajiura et al., 2015). Furthermore, the ulcer reduction rate was 94.4% in the control group, consistent with values of 84.5%–97.2% reported in other studies of PPI-treated patients (Takeuchi et al., 2011; Takayama et al., 2013; Ko et al., 2019). The ulcer reduction rate was significantly higher in the gel group than in the control group (97%). Large-sized resections correlated with a low ulcer healing rate. This study permitted different kinds of PPI except for potassium-competitive acid blockers because a previous study confirmed that the healing speed of ESD-induced artificial ulcers was not affected by the CYP2C19 genotype (Yoshizawa et al., 2016).

Delayed bleeding occurs in up to 9% of patients who undergo gastric ESD in clinical practice (Park et al., 2011; Lian et al., 2012; Facciorusso et al., 2014). Although the present study demonstrated a decreasing trend in the gel group, the delayed bleeding rate (active hemorrhage on endoscopic examination) post-ESD did not differ significantly between the control (*n* = 5, 8.1%) and gel (*n* = 1, 1.6%) groups. One possible reason for this result might be the small number of patients in both groups. A recent RCT showed the PGA sheet could not reduce delayed bleeding after gastric ESD, but its authors indicated that the relatively conservative inclusion criteria might underestimate the function of PGA shielding in high-risk patients (Kataoka et al., 2019). Further large-scale studies are needed to establish the effects of the gel in individuals with an elevated risk of delayed bleeding, e.g., those taking dual antiplatelet or anticoagulant medications.

Bio-sheets and PGA sheets with fibrin glue have been shown to increase the healing rate in artificial ulcers or decrease complications post-ESD (Kwon et al., 2015; Sakaguchi et al., 2015), but a recent randomized controlled trial by Kataoka et al. (2019) showed that PGA sheets could not reduce delayed bleeding after gastric ESD. Unfortunately, the above approaches are complex and time-consuming: the mean procedure time for applying the sheets and glue was 20.4 ± 9.5 min (Tsuji et al., 2015). Furthermore, the shielding method using fibrin glue has additional disadvantages, such as infection risk.

Self-assembling peptides (SAPs) are small oligopeptides generally containing repeated amino acid sequences that can spontaneously self-assemble to form molecules with distinct nanostructural properties (Lee et al., 2019). SAPs that function like natural extracellular matrix have been used in gastrointestinal endoscopic surgery to prevent perioperative bleeding (Subramaniam et al., 2021) and delayed bleeding (Soons et al., 2021). Several studies showed the hemostatic utility of SAPs following endoscopic mucosal resection or ESD for diverse gastrointestinal lesions (Yoshida et al., 2014; Pioche et al., 2016; Subramaniam et al., 2019). Hydrogels are also being developed and show promising results (Miura et al., 2021). In addition to having a hemostatic function, SAPs were reported to facilitate ulcer healing within the first week and increase the scarring rate at 4 and 8 weeks after ESD (Uraoka et al., 2016). Our previous preclinical study assessed the stability of the novel gel in a porcine model (Li et al., 2021). We found that the gel could be applied to an ulcer under endoscopic guidance in only 36.50 ± 6.21 s, and the gel appeared to remain on six of eight treated ulcers for approximately 1 week, which should be sufficient to shield an ulcer from the gastric medium during initial healing (Li et al., 2021). The present clinical trial showed that 23 of 46 lesions still had gel coverage 3–5 days after ESD, possibly due to differences between humans and pigs in anatomical structure and physiological characteristics. More interestingly, the gel could significantly reduce the proportion of the A1 stage in the first 3–5 days after ESD, which means that in the early healing phase of artificial ulcers, the use of the gel might be critical to protecting the ulcer floor from gastric secretions. The classification system proposed by Sakita (Sakita, 1972) is an available guide for the clinical treatment and prognosis of ulcers, but it is not objective or continuous. Moreover, the distinction between A- and H-stage ulcers is not clear. Hence, whether the conventional ulcer classification system was suitable to evaluate post-ESD ulcers in this study still needs discussion.

This study had some limitations. First, although it was a multicenter, open-label trial, the relatively small number of patients may have underpowered the study to detect real differences between groups in some comparisons, such as the incidence of delayed bleeding. Second, the ulcer reduction rate outcome used the resected specimen to calculate the initial ulcer size. However, the size of the resected tissue may have been smaller than that of the actual ulcer surface because of tissue shrinkage after cutting and electrocoagulation during ESD. Therefore, the ulcer reduction rate might have been overestimated. Nonetheless, it does not invalidate the comparisons between groups since the same method was used for both groups. Third, delay bleeding rate was not significantly different between groups; it might be due to the limited sample size of this study. All patients underwent endoscopic hemostasis of the wound. The reason for the relatively high bleeding rate of this study might be because of the different definitions of delayed bleeding. Indeed, in this study, a patient with minor bleeding was defined as the presence of hematemesis, melena, or a decrease in hemoglobin ≥ 2 g/dl, with blood clots and exposed vessels observed during endoscopy. On the other hand, in many other studies, the definition of minor bleeding also includes endoscopic signs of bleeding (Goto et al., 2012; Toyokawa et al., 2012). Finally, 17 patients were lost to follow-up; this relatively high dropout rate may have influenced the reliability of the main outcome.

In conclusion, the results of this open-label, randomized, multicenter trial indicate that applying a new self-assembling gel to an artificial gastric ulcer produced by ESD facilitated healing during the first 28 days. Although the present study demonstrates a decreasing trend in the gel group, it did not unequivocally show that it prevented ESD-induced delayed bleeding. Nonetheless, this study suggests that the self-assembling gel is safe and effective for accelerating gastric ulcer healing of iatrogenic gastric ulcers after ESD.

## Data Availability

The original contributions presented in the study are included in the article/Supplementary Material, further inquiries can be directed to the corresponding author.
