# Supplementary material for: Effectiveness and safety of a newly designed self-assembling gel in the treatment of endoscopic submucosal dissection-induced gastric ulcer: A multicenter randomized controlled trial
Source: Front Pharmacol. 2022 Dec 1;13:1002381. doi: 10.3389/fphar.2022.1002381 (PMC9751593; doi:10.3389/fphar.2022.1002381)
Supplement: Supplementary file 1 [file Table1.DOCX]

**Supplementary Table 1. Comparison of vital signs and laboratory tests before and after endoscopic submucosal dissection (ESD)**

| Characteristics | Pre-ESD | | | 3–5 days after ESD | | | 28 days after ESD | | |
| --- | --- | --- | --- | --- | --- | --- | --- | --- | --- |
|  | Control group (*n* = 62) | Gel group (*n* = 63) | *P* | Control group (*n* = 62) | Gel group (*n* = 60) | *P* | Control group (*n* = 56) | Gel group (*n* = 53) | *P* |
| Systolic BP (mmHg) | 129.4±16.1 | 127.3±16.4 | 0.477 | 120.7±11.8 | 121.4±13.0 | 0.759 | 123.6±9.8 | 126.0±11.0 | 0.234 |
| Diastolic BP (mmHg) | 76.7±11.1 | 78.0±10.6 | 0.492 | 70.3±8.7 | 74.2±9.8 | 0.021 | 72.2±6.0 | 75.4±7.5 | 0.014 |
| Heart rate (times/min) | 78.3±11.4 | 80.1±14.3 | 0.441 | 70.8±9.7 | 70.7±9.6 | 0.935 | 70.7±7.1 | 70.7±6.6 | 0.977 |
| Body temperature (kelvin) | 309.9 (0.3) | 309.9 (0.6) | 0.668 | 309.9±0.34 | 310.0±0.34 | 0.138 | 310.1 (0.4) | 310.1 (0.5) | 0.714 |
| Breathing rate (times/min) | 18 (1.0) | 18 (0.0) | 0.126 | 18.0 (0) | 18.0 (0) | 0.923 | 18.0 (0) | 18.0 (0) | 0.941 |
| Abdominal pain (VAS) |  |  |  |  |  | 0.203 |  |  | >0.999 |
| 0 |  |  |  | 52 (83.9) | 49 (77.8) |  | 56 (100) | 53 (100) |  |
| 1 |  |  |  | 6 (9.7) | 13 (20.6) |  |  |  |  |
| 2 |  |  |  | 3 (4.8) | 1 (1.6) |  |  |  |  |
| 3 |  |  |  | 1 (1.6) | 0 (0.0) |  |  |  |  |
|  | *n* = 61 | *n* = 62 |  |  |  |  | *n* = 49 | *n* = 45 |  |
| WBC count (×10^9^/L) | 5.24±1.35 | 5.67±1.85 | 0.144 |  |  |  | 5.22±1.31 | 5.31±1.75 | 0.776 |
| Red cell count (×10^9^/L) | 4.38±0.44 | 4.46±0.58 | 0.451 |  |  |  | 4.42±0.49 | 4.57±0.61 | 0.187 |
| Lymphocytes (×10^9^/L) | 1.65±0.52 | 1.71±0.55 | 0.587 |  |  |  | 1.71±0.53 | 1.67±0.53 | 0.667 |
| Hemoglobin content (g/L) | 133.1±21.2 | 136.5±13.9 | 0.283 |  |  |  | 134.8±13.6 | 136.8±16.7 | 0.521 |
| Platelet count (×10^9^/L) | 194.9±49.1 | 198.3±50.1 | 0.708 |  |  |  | 187.1±44.7 | 196.1±52.5 | 0.368 |
| Neutrophil count (×10^9^/L) | 3.0 (1.65) | 3.4 (1.26) | 0.689 |  |  |  | 3.02±1.04 | 3.16±1.50 | 0.588 |
|  | *n* = 59 | *n* = 60 |  |  |  |  | *n* = 48 | *n* = 46 |  |
| GGT (U/L) | 18.0 (15.0) | 17.0 (13.3) | 0.960 |  |  |  | 17.5 (13.0) | 20.0 (17.5) | 0.705 |
| ALT (U/L) | 17.0 (10.0) | 15.0 (8.0) | 0.209 |  |  |  | 14.0 (10.0) | 14.5 (120) | 0.919 |
| AST (U/L) | 18.0 (7.0) | 17.0 (8.0) | 0.368 |  |  |  | 19.0 (6.0) | 19.5 (6.3) | 0.705 |
| Creatinine (µmol/L) | 66.0 (19.0) | 68.0 (24.8) | 0.903 |  |  |  | 72.7±13.3 | 69.4±14.9 | 0.266 |
| BUN (mmol/L) | 5.20 (1.80) | 5.10 (1.85) | 0.901 |  |  |  | 4.5 (2.83) | 4.5 (1.62) | 0.558 |
| Total bilirubin (µmol/L) | 11.5 (6.4) | 11.7 (5.6) | 0.928 |  |  |  | 12.0 (5.4) | 11.1 (6.0) | 0.325 |
| Total phosphorus (g/L) | 67.7±6.5 | 68.5±5.6 | 0.490 |  |  |  | 71.8±4.1 | 71.5±4.3 | 0.709 |
|  | *n* = 62 | *n* = 61 |  |  |  |  | *n* = 48 | *n* = 44 |  |
| APTT (s) | 29.6 (6.12) | 28.5 (5.55) | 0.276 |  |  |  | 28.7 (5.88) | 28.6 (4.68) | 0.611 |
| Prothrombin time (s) | 11.1 (1.83) | 11.2 (1.50) | 0.962 |  |  |  | 11.2 (1.3) | 11.0 (1.63) | 0.549 |
| INR | 0.93±0.06 | 0.93±0.07 | 0.733 |  |  |  | 0.93±0.05 | 0.92±0.06 | 0.763 |
| Thrombin time (s) | 17.9 (1.35) | 18.0 (1.30) | 0.640 |  |  |  | 18.0±0.89 | 17.9±0.93 | 0.631 |
| D2 polymers (mg/L) | 0.23 (0.22) | 0.23 (0.22) | 0.674 |  |  |  | 0.31 (0.35) | 0.31 (0.29) | 0.825 |
| Fibrinogen (g/L) | 2.89 (0.59) | 2.79 (0.67) | 0.360 |  |  |  | 2.84 (0.71) | 2.98 (0.57) | 0.606 |
|  | *n* = 40 | *n* = 44 |  |  |  |  | *n* = 15 | *n* = 5 |  |
| Fecal leukocytes (+) | 0 (0) | 0 (0) | >0.999 |  |  |  | 0 (0) | 0 (0) | >0.999 |
| Fecal erythrocytes (+) | 0 (0) | 0 (0) | >0.999 |  |  |  | 0 (0) | 0 (0) | >0.999 |
| Fecal occult blood test (+) | 9 (22.5) | 8 (18.2) | 0.787 |  |  |  | 9 (60.0) | 2 (40.0) | 0.617 |
