# Supplementary material for: Effectiveness and safety of a newly designed self-assembling gel in the treatment of endoscopic submucosal dissection-induced gastric ulcer: A multicenter randomized controlled trial
Source: Front Pharmacol. 2022 Dec 1;13:1002381. doi: 10.3389/fphar.2022.1002381 (PMC9751593; doi:10.3389/fphar.2022.1002381)
Supplement: Supplementary file 2 [file Table2.DOCX]

[**Supplementary**](javascript:;) **Table 2. Ulcer healing at 3–5 days and 28 days after endoscopic submucosal dissection (ESD) before multiple interpolations**

| Characteristics | Full analysis set | | | Per protocol set | | |
| --- | --- | --- | --- | --- | --- | --- |
|  | Control group | Gel group | *P* | Control group | Gel group | *P* |
|  | (*n*=62) | (*n*=63) |  | (*n*=53) | (*n*=50) |  |
| Ulcer area (mm^2^), median (IQR) |  |  |  |  |  |  |
| Initial | 763.4 (514.2) | 868.7 (724.6) | 0.371 | 750.2 (533.6) | 868.7 (744.4) | 0.259 |
|  | N=61 | N=62 |  | N=53 | N=49 |  |
| 28 days after ESD | 33.9 (50.8) | 15.3 (40.4) | 0.012 | 34.7 (50.0) | 15.4 (43.9) | 0.019 |
|  | N=55 | N=52 |  | N=53 | N=50 |  |
| Ulcer reduction rate at 28 days |  |  |  |  |  |  |
| All patients, mean±SD | 94.6± 5.0 | 97.0±4.0 | 0.007 | 94.5±5.0 | 96.8±4.0 | 0.009 |
|  | N=54 | N=52 |  | N=53 | N=50 |  |
| No gel coverage at 3–5 days, median (IQR) |  | 98.1 (3.37) |  |  | 98.0 (3.21) |  |
|  |  | N=24 |  |  | N=23 |  |
| [Minority gel coverage at 3–5 days, median (IQR)](javascript:void(0);) |  | 96.5 (4.52) |  |  | 96.1 (4.89) |  |
|  |  | N=18 |  |  | N=17 |  |
| Majority gel coverage at 3–5 days, median (IQR) |  | 99.9 (1.63) | 0.076 |  | 99.9 (1.63) | 0.044 |
|  |  | N=6 |  |  | N=6 |  |
| Ulcer stage at 3–5 days, *n* (%) |  |  | <0.001 |  |  | <0.001 |
| A1 stage | 43 (70.5) | 14 (24.1) |  | 36 (67.9) | 11 (23.9) |  |
| A2 stage | 18 (29.5) | 43 (74.1) |  | 17 (32.1) | 34 (73.9) |  |
| H1 stage | 0 (0) | 1 (1.7) |  | 0 | 1 (2.2) |  |
| Ulcer stage at 28 days, *n* (%) |  |  | 0.143 |  |  | 0.206 |
| A2 stage | 6 (10.7) | 5 (9.4) |  | 6 (11.3) | 5 (10.0) |  |
| H1 stage | 37 (66.1) | 33 (62.3) |  | 36 (67.9) | 32 (64.0) |  |
| H2 stage | 9 (16.1) | 4 (7.5) |  | 8 (15.1) | 4 (8.0) |  |
| S1 stage | 4 (7.1) | 11(20.8) |  | 3 (5.7) | 9 (18.0) |  |
| Delayed bleeding, *n* (%) | 8 (12.9) | 6 (9.5) | 0.549 | 7 (13.2) | 5 (10.0) | 0.761 |
